# Correspondence of biological condition models of California streams at statewide and regional scales

**DOI:** 10.1007/s10661-014-4086-x

**Published:** 2014-11-11

**Authors:** Jason T. May, Larry R. Brown, Andrew C. Rehn, Ian R. Waite, Peter R. Ode, Raphael D. Mazor, Kenneth C. Schiff

**Affiliations:** 1 United States Geological Survey, California Water Science Center, Sacramento, CA USA; 2 Aquatic Bioassessment Laboratory, Water Pollution Control Laboratory, California Department of Fish and Wildlife, Rancho Cordova, CA USA; 3 United States Geological Survey, Oregon Water Science Center, Portland, OR USA; 4 Southern California Coastal Water Research Project, Costa Mesa, CA USA

**Keywords:** Bioassessment, Macroinvertebrates, O/E, Monitoring, Boosted regression trees, Planning, Conservation, Spatial scale

## Abstract

We used boosted regression trees (BRT) to model stream biological condition as measured by benthic macroinvertebrate taxonomic completeness, the ratio of observed to expected (O/E) taxa. Models were developed with and without exclusion of rare taxa at a site. BRT models are robust, requiring few assumptions compared with traditional modeling techniques such as multiple linear regression. The BRT models were constructed to provide baseline support to stressor delineation by identifying natural physiographic and human land use gradients affecting stream biological condition statewide and for eight ecological regions within the state, as part of the development of numerical biological objectives for California’s wadeable streams. Regions were defined on the basis of ecological, hydrologic, and jurisdictional factors and roughly corresponded with ecoregions. Physiographic and land use variables were derived from geographic information system coverages. The model for the entire state (*n* = 1,386) identified a composite measure of anthropogenic disturbance (the sum of urban, agricultural, and unmanaged roadside vegetation land cover) within the local watershed as the most important variable, explaining 56 % of the variance in O/E values. Models for individual regions explained between 51 and 84 % of the variance in O/E values. Measures of human disturbance were important in the three coastal regions. In the South Coast and Coastal Chaparral, local watershed measures of urbanization were the most important variables related to biological condition, while in the North Coast the composite measure of human disturbance at the watershed scale was most important. In the two mountain regions, natural gradients were most important, including slope, precipitation, and temperature. The remaining three regions had relatively small sample sizes (*n* ≤ 75 sites) and had models that gave mixed results. Understanding the spatial scale at which land use and land cover affect taxonomic completeness is imperative for sound management. Our results suggest that invertebrate taxonomic completeness is affected by human disturbance at the statewide and regional levels, with some differences among regions in the importance of natural gradients and types of human disturbance. The construction and application of models similar to the ones presented here could be useful in the planning and prioritization of actions for protection and conservation of biodiversity in California streams.

## Introduction

Threats to aquatic systems are increasing in number and severity as world population and associated resource demands increase (Postel and Richter 2003; Dudgeon et al. 2006; Strayer and Dudgeon 2010). Developing methods to adequately protect and restore the biological integrity of flowing waters with limited financial resources is a challenge faced by water resource agencies worldwide (Novotny et al. 2004; Southerland et al. 2008). There is a growing need for tools to inform managers and aid them in understanding and balancing these conflicting resource demands. Environmental planners and managers must utilize many sources of information, including empirical and modeled data, to better understand complex relations and then formulate sound management (Barmuta et al. 2011).

California is the third largest state in the USA (423,970 km^2^) and has the greatest population (over 37 million as of 2011). The population is continuing to grow and is expected to reach 48 million by 2025 (Public Policy Institute of California. Digital data and accessed 2012). California is also geographically and biologically diverse, including landscapes varying from high mountains to deserts to temperate rainforest. The impacts of human development, such as mining, ranching, timber harvest, urbanization, and agricultural land use, on California rivers and streams have been well documented (Mount 1995; Moyle 2002). As the demand for municipal, industrial, and agricultural water supply increases, the difficulty of maintaining instream conditions for protection and management of natural ecosystems will also increase. To guide efficient coordination among California’s resource agencies, these agencies need tools that provide a broad overview of relationships between land use activities and beneficial uses of aquatic systems.

Bioassessments are now routinely used in resource management, and many regulatory agencies have implemented or are now moving toward implementation of quantitative, enforceable biocriteria (Barbour et al. 2000). Stream bioassessments, including individual metrics, multimetric indices, and other measures of aquatic assemblages, have successfully been used as indicators of stream biological condition throughout the world (e.g., Barbour et al. 1999; Ode et al. 2005a; Bonada et al. 2006; Hering et al. 2006). In California, state and federal resource agencies have been sampling benthic macroinvertebrate assemblages for over two decades (Tetra Tech 2003). As part of these efforts, extensive GIS information has been assembled for all sampled watersheds (Ode et al. 2011).

The foundation of bioassessments is the reference condition approach (Hughes et al. 1986; Bailey et al. 2004). In this approach, test sites are compared to designated reference (least-impacted) sites; indicators for scoring these samples can be in the form of single metrics (e.g., taxonomic richness or richness of a particular group such as Coleoptera richness), multimetric indices (e.g., an index of biotic integrity), and/or scores on multivariate axes (e.g., an axis derived from a nonmetric multidimensional scaling axis aligned with a stressor gradient) of biological condition. A biological index commonly associated with the reference condition approach is the taxonomic completeness of sites, which is calculated as the ratio of taxa observed (O) to taxa expected (E) at a site if the site was least-impacted by human disturbance (i.e., reference condition). The list of taxa expected at a site is estimated from predictive models based on samples from reference sites (see “Methods” for details). This ratio of observed to expected (O/E) taxa has been widely implemented as an index of biological condition by state, federal, and international water management agencies (Linke and Norris 2003; Hawkins 2006; Ode et al. 2008; Aroviita et al. 2009; Carlisle et al. 2009). An O/E of 1.0 means the site’s taxa richness is equal to the average for the reference sites. O/E values near 1 imply high biological integrity and taxonomic completeness whereas O/E values less than 1 imply some loss of taxonomic completeness and degradation of biological integrity. Each tenth of a point below 1 suggests a 10 % loss of expected taxa. Because O/E is based on raw compositional data (i.e., the species list at a site compared to the list expected at a reference site with the same environmental conditions) rather than calculated metrics that may only apply to certain areas based on biogeography (e.g., Plecoptera are only expected in cool, well-oxygenated streams), it has the potential to serve as a comprehensive indicator of taxonomic completeness and inferred biotic condition (Hawkins 2006; Aroviita et al. 2010; Turak et al. 2011). O/E values have been used as a measure of conservation value of sites, in the context of protecting stream biodiversity. For example, Aroviita et al. (2010) found a positive association of O/E values with the presence of threatened macroinvertebrate species. Linke and Norris (2003) showed O/E to be effective in identifying areas that may need restoration, areas with high conservation potential, and areas where biodiversity loss has been so great that rehabilitation is not likely to be cost effective.

Understanding the factors generating patterns in bioassessment scores among a group of sites requires understanding of stressor-response relations. Such stressor-response relations have been of long-term interest to aquatic ecologists studying stream ecosystems (e.g., Allan 2004). The use of computer-based geographic information systems (GIS) has greatly increased the ability of aquatic ecologists to include considerations of land use, and the social and economic aspects of human habitation on the landscape, when exploring the effects of human disturbance on biological condition (Alberti et al. 2007; Falcone et al. 2010; Shandas and Alberti 2009; Turak et al. 2011; Clapcott et al. 2012). Thus, measurements at various spatial scales ranging from the regional climate to riparian habitat in a specific stream reach can be used to understand the relations of the environment with the biotic communities (Sponseller et al. 2001; Weigel et al. 2003; Allan 2004).

Ecologists are trying to understand the spatial scales and processes associated with human and natural disturbances that affect aquatic biota (e.g., Allan 2004). Models provide a useful framework for testing hypotheses, determining potential direct and indirect linkages, and directing where further research is needed (Van Sickle et al. 2004; Ode et al. 2008; Waite et al. 2010, 2012, 2014). When developing models, there is often a question about the appropriate spatial scale for model development. There is an expectation that regionally specific models will perform better than models based on larger spatial scales. Models at smaller spatial scales typically allow for greater insight regarding disturbance-related variables that are likely to operate at watershed and site-specific scales (Clapcott et al. 2012; Waite et al. 2012). However, larger scale models may sometimes be more desirable to land managers because they simplify the development of management strategies over larger geographic areas. They may also be less costly to develop given that developing models for separate regions would require sampling more sites than for a single larger area.

Advances in modeling techniques, including neural networks (Cereghino et al. 2003; Olden et al. 2006; Feio and Poquet 2011), structural equation models (Shipley 2000; Hermoso et al. 2011), Bayesian networks (Webb and King 2009; Feio and Poquet 2011), and classification and regression tree analysis (CART) or extensions of this approach (Breiman et al. 1984; De’ath and Fabricius 2000; Prasad et al. 2006; De’ath 2007), have greatly improved the ability to model the highly heterogeneous, non-normal and non-linear types of data common in environmental assessments (e.g., Waite et al. 2010, 2012). Boosted regression trees (BRT) are an extension of CART (Aertsen et al. 2010). CART is a simple but robust analytical technique well suited to multivariate ecological data containing complex levels of information (De’ath and Fabricius 2000) but can suffer from poor predictive capabilities (De’ath 2007) because it develops a single parsimonious model that overfits the data. Boosting improves model structure and predictive performance by fitting many simple models from samples of the data and combining them to better estimate the response. BRT models require few assumptions regarding data distributions compared with traditional modeling techniques such as multiple linear regression (MLR) and general additive models (GAM) and have been consistently shown to perform better (i.e., higher *R* ^2^ values) than traditional techniques such as MLR and other tree-based methods (e.g., Aertsen et al. 2010; Waite et al. 2010, 2012, 2014).

Our primary objective in this paper was to construct boosted regression tree models of the response of the O/E index of macroinvertebrate taxa to a set of explanatory variables developed from GIS measures of land cover and land use. The purpose of constructing these models is to develop an understanding of patterns in taxonomic completeness at statewide and regional scales in relation to environmental measures. We are particularly interested in determining if the environmental variables related to taxonomic completeness in the statewide model are similar to those in the regional models. We compare results from our statewide model to the results from each regional model to assess representativeness of a single statewide model. Our results have implications for the ongoing development of numerical biological objectives for California streams (Ode et al. 2011; SWAMP 2012). Additionally, models similar to ours could potentially aid California resource management agencies in the prioritization of future monitoring and conservation efforts for stream aquatic resources.

## Methods

### Regions for modeling

**Fig. 1:**
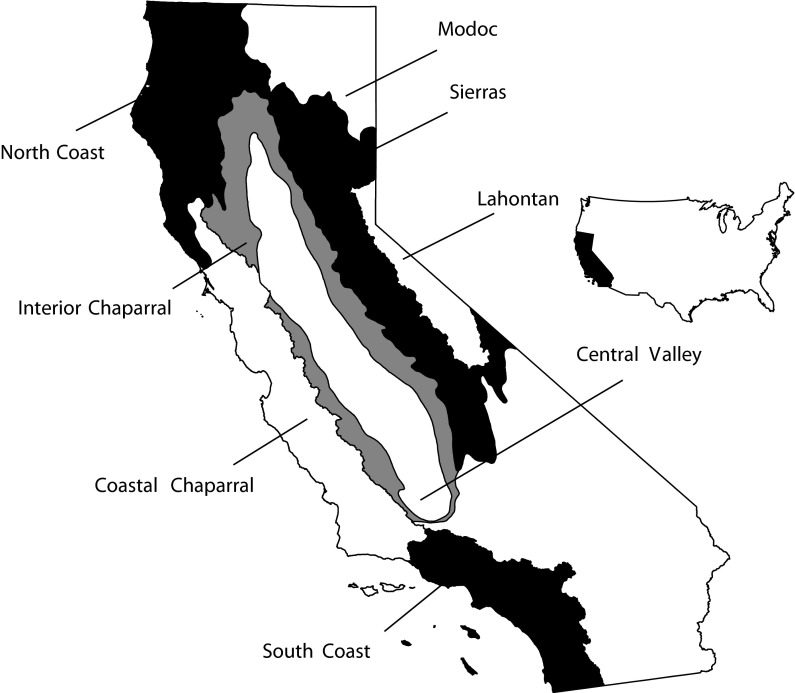
Map of California with Perennial Stream Assessment (PSA) regions

The regions used for this study were defined on the basis of ecological, hydrologic, and jurisdictional factors and were developed as reporting units for California’s ambient Perennial Stream Assessment (PSA) program (Fig. 1; Ode et al. 2011). The state includes eight PSA regions that roughly correspond with Omernik Level III ecoregions (Omernik 1987; Ode et al. 2011): Northern Coastal California (herein referred to as North Coast), Western Sierra Mountains (herein referred to as Sierras), Central Lahontan (herein referred to as Lahontan), Modoc and Deserts (herein referred to as Modoc), Coastal Chaparral, Interior Chaparral, the Central Valley, and Southern Coastal California (herein referred to as South Coast).

### Environmental variables

The watershed upstream of each site was delineated using USGS 7.5-minute quadrangle digital raster graphics (DRG) as base layers. The DRGs were overlaid with National Hydrography Dataset (NHD) medium resolution stream lines (U.S. Geological Survey 2007). Watershed boundaries were digitized at a scale of (1:100). Adjacent watershed polygons were edge matched to eliminate all overlaps and gaps. All work was conducted using ArcGIS, ArcMap 9.2 (Environmental Systems Research Institute, Redlands, CA) GIS software. Landscape variables, including measures of elevation, slope, climate, land cover and land use based on National Land Cover Data 2001 (NLCD 2001); population density; road networks; hydrology; and dams were determined for each watershed and local 1 and 5-km areas from available national and regional datasets (Table 1, Appendix 1). Local climate (i.e., precipitation and temperature measures) was determined from Parameter-elevation Relationships on Independent Slopes Model (PRISM) databases (Daly et al. 2008; PRISM 2010). The NLCD (2001) was utilized rather than NLCD (2006) because it was the data available to us at the time the calculations were being done. It is unlikely that this choice affects our results. The biological data were collected over a time period (1999–2009) including both years. Also correlations of metrics calculated from both data sets were quite high, ranging from 0.93 to 0.99.

**Table 1:** Model variable codes, units, scale, and description. Scale refers to spatial area of analysis (point, at the collection; 1 km, watershed area within 1 km of site; 5 km, watershed area within 5 km of site; WS, upstream watershed area)

		Scale				
Variable code	Unit	Point	1 km	5 km	WS	Description
Response variables-invertebrate metrics						
O/E	Ratio	X				Ratio of number of observed taxa at a site to the expected taxa based on modeled reference sites, all taxa
O/E_50_	Ratio	X				Ratio of number of observed taxa at a site to the expected taxa based on modeled reference sites, common taxa with a probability of capture at 50 % sites
Explanatory variables						
Temp	°C	X				30-year (1971–2000) average max temperature
PPT	mm	X				30-year (1971–2000) average annual precipitation
Slope	%	X				Gradient at reach level
Elevation	m	X				Elevation at site
DamDist	m					Distance to nearest upstream dam in catchment
Area	km^2^				X	Watershed area
Ag	%		X	X	X	Percent agricultural lands (row crop, pasture)
AgUrb21	%		X	X	X	Percent developed land (urban, row crop, pasture, nlcd class 21)
Burns20052009	#		X			Number of burns between 2005 and 2009
CalPipe100kPer	%				X	Percent canals or pipes at the 100-k scale
CanalPipeDist100k	m				X	Total length of FTYPE equal to Canal Ditch and Pipeline in NHD+
CaO	%				X	Percent calcite mineral content
CODE_21	%		X	X	X	Percentage of urban/recreational grass (nlcd 21)
CondQrm	μS/cm				X	Predicted electric conductivity (Olson and Hawkins 2012)
DamCount	#		X	X	X	Number of dams
DamDensArea	dams/km^2^		X	X	X	Density of dams, by area
DamStorage	km2				X	Total dam storage
GravelMines	#		X	X	X	Count of gravel mines
GravelMinesDens	gravel mines/km^2^		X	X	X	Density of gravel mines
Grazing	%		X	X	X	Percent of area allotted to grazing on USFS and BLM lands
IMPERVMEAN	%		X	X	X	Impervious surfaces from NLCD
MAFLOWU	cfs				X	Cumulative annual discharge in NHD+, unit area method
MgO	%				X	Percent magnesium oxide mineral content
Mines	#		X	X	X	Count of mines
MinesDens	mines/km^2^		X	X	X	Density of mines
Ngeo	%				X	Percent nitrogen geology
NewAg	%		X	X		Percent new ag (nlcd class 36, 46, or 56)
NewUrb	%		X	X		Percent new urban (nlcd class 32, 42, 52, or 62)
PctSed	%				X	Percent sedimentary geology
Pop	# people/km^2^		X	X	X	Population density in 2000
rDDENSC12	roads/km2		X	X	X	Total density of paved roads (nlcd class 1 and 2)
rDDENSC123	roads/km2		X	X	X	Total density of paved and unpaved roads (nlcd classes 1, 2, and 3)
STREAMORDER	#				X	Strahler stream order
URBAN	%		X	X	X	Percentage of polygon designated as urban (nlcd class 22, 23, 24)

Subwatershed variables were derived at 1 and 5-km scales. The intersection of the watershed area with a circle of 1 or 5 km radius around the site defined the portion of the watershed draining to the site. We chose these three spatial scales (watershed, 5-km scale, and 1-km scale of land cover) based on preliminary analysis of five spatial scales, the three used in this paper plus measures of riparian buffer at 1-km, 5-km, and watershed-wide scales. The buffer measures were not included in the final variable list because we had less confidence in the calculations because pixel size and distribution in stream corridors made definition of a uniform buffer difficult. The errors introduced were especially important for smaller streams. Although other choices for the circle radius are certainly possible, 1 and 5 km seemed a reasonable estimate of “nearby” influences on a stream, especially given that larger values would begin encompassing large percentages of some of the smaller watersheds.

While some reach level habitat and water quality information was available for a portion of our sites, we chose not to include these data in our analysis since they were not available for all sites. This is not to imply that reach-scale data would not result in better models; however, the loss in sample size would have been substantial and limited our ability to model the separate regions.

Because basic water chemistry was not consistently collected at all sites, we used the method of Olson and Hawkins (2012) to predict baseline electrical conductivity (CondQrm) values as an indicator of reach-scale water chemistry. We do not include CondQrm as a prediction of actual conductivity but intend it to act as a gross indicator of water quality based on the environmental setting (e.g., saline desert stream versus low conductivity alpine stream). Briefly, the model uses GIS data on atmospheric deposition, geology, climate, soil, topography, vegetation, and groundwater influences to predict electrical conductivity. The model was developed and validated based on an extensive data set of reference quality sites from the western USA, including California (Olson and Hawkins 2012). The correlation between CondQrm and measured specific conductance was relatively high (rho = 0.8) at the subset of sites where water quality measurements were available.

### Macroinvertebrate data collections

The data set consisted of 1,386 macroinvertebrate samples collected during 1999–2009. The number of sampling sites by region with percentage of sites classified as reference sites in parenthesis are as follows: North Coast = 197 (28 %), Sierras = 203 (58 %), Lahontan = 200 (59 %), Modoc = 56 (43 %), Coastal Chaparral = 216 (20 %), Interior Chaparral = 75 (40 %), Central Valley = 54 (2 %), and the South Coast = 385 (28 %). Reference sites were determined using screening procedures based on thresholds for categories of land use and land cover and select reach level habitat information (Peter Ode, California Department of Fish and Wildlife, unpublished data). While the distribution of sample sizes and reference sites would ideally be equally distributed across all regions, at the time of the analysis these were the “best” available data for a regional bioassessment analysis. Future analyses efforts will likely have larger sample sizes and more complete reach level data as bioassessments continue.

This data set was compiled from multiple state and federal studies, which used comparable targeted-riffle and reach-wide sampling protocols (see Ode et al. 2005a; Rehn et al. 2008; Ode et al. 2011 for details on combining data sets). The agencies included eight California Regional Water Quality Control Boards (North Coast, Central Valley, Lahontan, San Francisco Bay, Central Coast, Santa Ana, Los Angeles, and San Diego), the US Environmental Protection Agency (EMAP Program), and the US Forest Service. The US Forest Service and most of the Regional Boards used a targeted-riffle protocol (sample area of 0.74 or 0.84 m^2^, respectively) (Harrington 1999 and Hawkins et al. 2001, respectively). EMAP data were collected with a reach-wide protocol (1.02 m^2^ sampled) (Peck et al. 2006). Rehn et al. (2007) determined that targeted-riffle and reach-wide methods gave comparable results for calculation of multimetric indices and O/E.

All collection efforts used D-frame kick nets with 500 micron mesh. Taxonomic composition was based on counts of 500 organisms per sample. Taxonomic classifications were generally to genus for insects except Chironomidae, which were only identified to family (Richards and Rogers 2006). Other organisms were identified to the lowest possible taxon, generally family or higher. A few taxa (e.g., worms and ostracods) were arbitrarily left at higher levels because of logistical constraints on processing time. O/E calculations were based on a randomly selected subset of 300 organisms from the original 500 count after removing ambiguous taxa.

### Calculation of O/E and O/E_50_

The O/E modeling approach has four main steps: (1) reference sites are classified according to degree of taxonomic similarity; (2) environmental variables associated with each class are identified; (3) discriminant function analysis is used to predict class membership of new test sites based on the values of their environmental predictor variables; (4) the observed list of taxa is compared to the expected list to calculate the O/E ratio (Hawkins et al. 2000).

O/E values were calculated from three models developed for areas of the state with different climatic and physiographic conditions, primarily based on mean basin elevation, mean annual precipitation, and air temperature (Ode et al. 2011). The number of taxa observed was obtained from samples collected at each site and the number of taxa expected at each site was modeled (Ode et al. 2008). Two O/E values were calculated for each site: O/E based on taxa with probability of capture (PC) >0, and O/E_50_ based on taxa with PC >0.5. The latter value reduces variability associated with rare species. Ostermiller and Hawkins (2004) discuss the statistical and biological reasons why use of an intermediate PC such as 0.5 may have advantages over the inclusion of all taxa. The exclusion of rare taxa associated with “data noise” helps with improving modeling accuracy and performance (Van Sickle et al. 2007).

As a check on using O/E as an indicator of stream biological condition, we correlated O/E and O/E_50_ scores with regional Index of Biotic Integrity (IBI) scores (Ode et al. 2005b; Rehn et al. 2005; Rehn et al. 2008; Herbst and Silldorff 2009) and commonly used macroinvertebrate community metrics. Pearson product–moment correlations of O/E and O/E_50_ with regional IBI were 0.7 and 0.6, respectively; with Ephermeroptera, Plecoptera, and Trichoptera (EPT) taxa richness were 0.8 and 0.7, respectively; with Coleoptera taxa richness were 0.5 and 0.4, respectively; with predator taxa richness were 0.8 and 0.6, respectively; with percent EPT taxa richness were 0.6 and 0.6, respectively; with percent tolerant taxa richness were −0.5 and −0.5, respectively; and with percent non-insect taxa were −0.5 and −0.5, respectively. These moderate to high correlations of O/E and O/E_50_ with other measures of biological condition support the assertion that O/E and O/E_50_ are good overall indicators of taxonomic completeness and biological integrity within our data set.

### Modeling approach

As previously explained, we developed models for the entire state and for each region. Our response variables were O/E and O/E_50_, and the explanatory variables consisted of several landscape land cover and land use variables and predicted electrical conductivity (CondQrm) (Table 1). The list of candidate variables was selected based on previous work in California (Ode et al. 2005a; Rehn et al. 2008; Waite et al. 2010, 2012, 2014; Brown et al. 2012). Although the O/E models were developed for regions with similar environmental settings and thus are expected to be relatively insensitive to natural gradients (Hawkins et al. 2000), we included several variables representing important natural gradients for verification (Table 1). While regions with lower sample sizes (i.e., *n* ≤ 75 sites) are less than ideal for BRT modeling, we chose to include these regions to provide statewide coverage. Because several regions had smaller sample sizes, we chose not to develop validated, predictive models. Such an approach requires splitting the available data into model development and model validation data sets. As a consequence, these models are limited to understanding the available data and are not appropriate for predicting O/E or O/E_50_ values at unsampled sites. Developing predictive models will become feasible as California’s bioassessment database continues to grow.

Regression trees are one technique within the commonly used CART or decision tree family, and their use and technical details have been described extensively in the literature (Breiman et al. 1984; De’ath and Fabricius 2000; Prasad et al. 2006). Trees attempt to explain variation in one categorical (classification) or continuous (regression) response variable by one or more explanatory variables. The resulting output is a dendrogram or “tree” with varying numbers of branches. Regression trees are developed following a hierarchical binary splitting procedure that attempts to find the best single explanatory variable that minimizes within group dissimilarity and maximizes between group dissimilarity in the response variable at each split. The CART model completes the process for each explanatory variable entered into the model and can thus determine the explanatory or predictive power of the variables. Regression trees have properties that are highly desirable for ecological data analysis: (1) they can handle numeric, categorical, and censored response variables (such as negative values censored to zero); (2) they are not affected by explanatory variables that follow non-normal distributions; and (3) they can model complex interactions simply (De’ath 2007). After the initial tree has been generated, BRT develops successive trees on reweighted versions of the data giving more weight to those cases that are incorrectly classified compared to those that are correctly classified. Thus, as more and more trees are developed, boosting increases the chance that cases that are difficult to classify initially are correctly classified in the final model. Overall, boosted trees retain the positive aspects of single CARTs and (1) improve predictive performance; (2) provide relative importance values for the explanatory variables; and (3) allow for testing and assessing the importance of nonlinearities and interactions (De’ath 2007). The BRT analysis was performed in the R statistical software (R Development Core Team 2007, version 2.10.0),) using the gbmplus (gbm, gradient boosting machine) library (Elith et al. 2008). Coefficient of determination (*R* ^2^) and the Akaike Information Criterion (AIC) were calculated to determine the most parsimonious model. AIC is used to assess the trade-off between the goodness of fit of the model and complexity of model in terms of the number variables included. For final models presented here, we provided *R* ^2^ and ΔAIC, where ΔAIC is the difference in AIC between the final best model (lowest AIC value) and any other model. If ΔAIC is 2 or less, models are not considered different.

We used untransformed data for predictor variables (Table 1), a bag fraction of 0.75 and a learning rate of 0.0001 for developing models. A bag fraction of 0.75 means that each tree is developed using a random selection of 75 % of the data. The learning rate influences the number of trees evaluated for the model; for our efforts, 10,000 trees was the maximum number of trees allowed for each model. Variable importance for each model was calculated using formulae developed by Friedman (2001) and implemented in the gbm library to estimate the relative influence of predictor variables. Variable importance is based on the number of times a variable is selected for splitting, weighted by the squared improvement to the models as a result of each split, averaged over all trees. The relative importance (or contribution) of each variable is scaled so that the sum adds to 100, with higher numbers indicating stronger influence on the response. We ran an initial model using all environmental variables (Table 1). We then deleted all variables with relative importance less than 5. The remaining 8–10 variables were used to develop the final BRT model. Additional reduction of variables is desirable because BRT techniques have a tendency to overfit models (Elith et al. 2008; Aertsen et al. 2010). The final model was selected by sequentially deleting variables and evaluating the effects on *R* ^2^ and AIC and examining partial dependency plots. Partial dependency plots are a graphical method of evaluating the effects of specific variables on the model. Partial dependency plots show the effect of a specific explanatory variable after accounting for the average effects of all other explanatory variables in the model (De’ath 2007; Elith et al. 2008).

### Regional applicability of the statewide model

We examined the applicability of a single statewide BRT model across all the PSA regions using the following process. We first calculated a predicted value of O/E for each site using the final statewide BRT model. We then regressed observed values of O/E against predicted values for each region and determined if the slope was significantly different from 1. We used *R* ^2^ and visual assessment of the distribution of regional values around the 1:1 line as indicators to determine if the statewide model provided unbiased predicted values for that region.

## Results

**Fig. 2:**
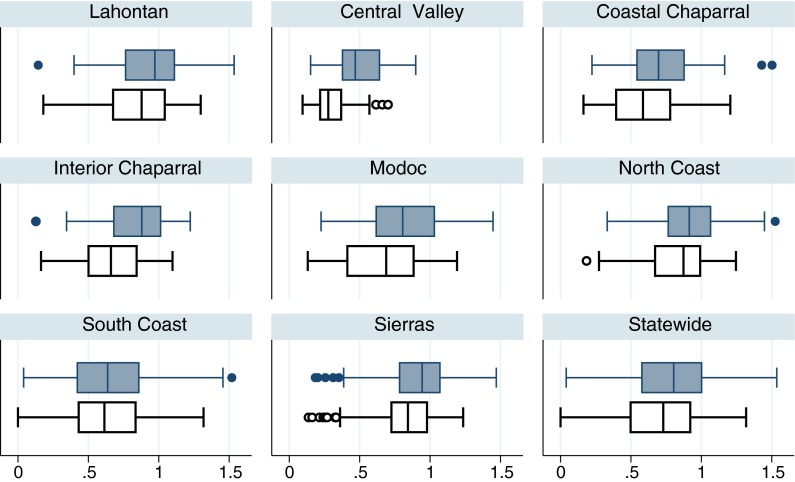
Boxplots of observed over expected (O/E) and (O/E_50_) values for statewide and for each PSA region. The *top, filled box plots* represent O/E and the *bottom, open box plots* represent O/E_50_ values

Climatic regimes, land use, and hydrology ranged widely among the PSA regions (Table 2). Median values of O/E_50_ were less than median values for O/E across the state and PSA regions (Fig. 2). The PSA regions with small sample size (*n* < 75) had the largest differences in median values, particularly the Central Valley.

**Table 2:** Median values of selected environmental variables for the state and by region. The minimum and maximum values are shown in parenthesis. For detailed variable descriptions, see Table 1 for variable codes: Area, AGURB21, Elevation, CondQrm, DamDensArea, IMPERVMEAN, MinesDens, and PPT, respectively

	Statewide (*n* = 1,386)	Lahontan (*n* = 200)	Central Valley (*n* = 54)	Coastal Chaparral (*n* = 216)	Modoc (*n* = 56)	Interior Chaparral (*n* = 75)	North Coast (*n* = 197)	South Coast (*n* = 385)	Sierras (*n* = 203)
Watershed area (km^2^)	43.2	89.1	103.9	320.6	468.4	112.8	28.7	48.8	39.8
	(0.12–40,509)	(1.1–2,307.1)	(5.8–31,185.2)	(1.79–8,722)	(1.81–8,812.4)	(2.94–575.9)	(1.1–40,509)	(0.12–4,118)	(1.2–4,565)
Watershed percent developed land (%)	2.03	0	30.13	5.45	0.46	2.06	2.16	5.75	0.06
	(0–100)	(0–45.27)	(0.34–100)	(0–80.78)	(0–41.98)	(0–63.73)	(0–83.93)	(0–100)	(0–34.38)
Elevation at sampling site (m)	638	2,037	28	102	1,361	379	535	459	1,433
	(0–3,107)	(1,258–3,107)	(3–141)	(0–1,269)	(0–2,205)	(66–940)	(3–1,753)	(0–2,246)	(512–2,997)
Percent canals or pipes at the 100 k scale (%)	0	0	9.28	0	0	0	0	0	0
	(0–68.65)	(0–17.24)	(0–68.65)	(0–47.39)	(0–17.99)	(0–54.64)	(0–19.02)	(0–60.89)	(0–54.31)
Predicted conductivity (μS/cm)	177	53	313	310	106	228	119	382	71
	(20–879)	(24–130)	(54–642)	(121–862)	(48–394)	(59–377)	(48–255)	(53–879)	(20–207)
Watershed dam density (number of dams/watershed area)	0	0	0	0	0	0	0	0	0
	(0–0.009)	0	(0–0.008)	(0–0.009)	(0–0.001)	0	(0–0.001)	(0–0.002)	(0–0.001)
Watershed impervious area (%)	0.150	0.104	1.700	0.167	0.092	0.179	0.050	0.367	0.112
	(0–57.55)	(0–11.10)	(0.07–51.81)	(0–32.93)	(0–12.89)	(0–18.56)	(0–32.96)	(0–57.55)	(0–10.17)
Watershed mine density (number of mines/watershed area)	0.018	0	0.042	0.024	0.004	0.049	0.015	0.026	0.009
	(0–10.25)	(0–0.62)	(0–1.69)	(0–1.38)	(0–1.50)	(0–2.48)	(0–0.75)	(0–0.99)	(0–10.25)
Annual precipitation (mm)	715	790	562	629	519	802	1,426	516	1,136
	(86–3,038)	(119–1,477)	(289–703)	(275–1,677)	(86–1,725)	(384–1,631)	(409–2,724)	(271–1,184)	(168–3,038)

**Fig. 3:**
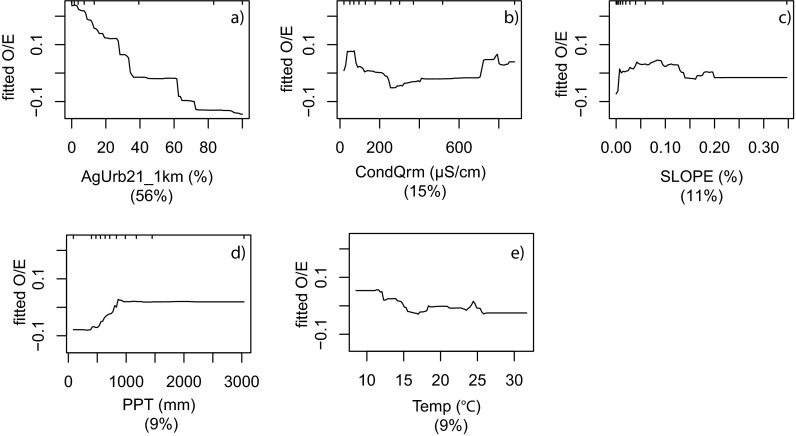
Partial dependency plots for the final statewide model (*n* = 1,386) of the response form of O/E (*y*-axis = fitted function of O/E) based on the effect of individual explanatory variables with the response of all other variables removed. Variables are shown in order of model importance: **a** percent developed land at 1 km scale (AGURB21_1km); **b** predicted conductivity (CondQrm); **c** slope; **d** average precipitation (PPT); and **e** average temperature (Temp). The *top tick marks* of each plot indicate deciles of the predictor variable

The best models included from 3 to 7 variables, with *R* ^2^ for O/E models ranging from 0.50 to 0.77 and *R* ^2^ for O/E_50_ models ranging from 0.44 to 0.84 (Table 3). The statewide models included five variables with the measure of human disturbance within 1 km (AGURB21_1km) having the highest variable importance by greater than a factor of three for the O/E model and greater by a factor of two for the O/E_50_ model. Less important variables included stream slope, predicted conductivity, and climate measures. Partial dependency plots for the statewide O/E model (Fig. 3) indicated an approximately linear decline in O/E as AGURB21_1km increased (Fig. 3a). The remaining variables exhibited highly variable patterns of response (Fig. 3b–e) and were likely important in fitting sites that were not associated strongly with AGURB21_1km.

**Table 3:** Boosted regression tree results by perennial stream assessment region for O/E and O/E_50_. The variables shown are those included in the final model with their relative importance shown in parentheses. The ΔAIC value represents the difference in AIC between the model shown and the next best model

Response variable	*R* ^2^	ΔAIC	Variables in order of influence (left to right)						
Statewide (*n* = 1386)									
O/E	0.50	27	AgUrb21_1km (56)	CondQrm (15)	SLOPE (11)	PPT (9)	Temp (9)		
O/E_50_	0.57	85	AgUrb21_1km (45)	SLOPE (19)	PPT (14)	CondQrm (12)	Temp (10)		
Coastal group									
Coastal Chaparral (*n* = 216)									
O/E	0.77	10	Pop_5km (31)	PPT (15)	Ag_WS (14)	CondQrm (13)	Elevation (12)	MgO (9)	MinesDens_WS (6)
O/E_50_	0.84	48	Pop_5km (33)	PPT (18)	Ag_WS (16)	CondQrm (12)	Elevation (11)	MgO (10)	
North Coast (*n* = 197)									
O/E	0.63	34	AgUrb21_WS (21)	Elevation (19)	Ngeo (19)	SLOPE (14)	MAFLOWU (14)	PPT (13)	
O/E_50_	0.76	24	AgUrb21_WS (30)	SLOPE (25)	MAFLOWU (18)	Ngeo (17)	PPT (10)		
South Coast (*n* = 385)									
O/E	0.68	83	UrBAN_1km (45)	MAFLOWU (16)	SLOPE (14)	CondQrm (13)	Temp (12)		
O/E_50_	0.59	21	UrBAN_1km (35)	SLOPE (16)	Ngeo (14)	Temp (13)	CondQrm (12)	MAFLOWU (10)	
Mountain group									
Lahontan (*n* = 200)									
O/E	0.65	6	SLOPE (33)	PPT (20)	MAFLOWU (16)	IMPERVMEAN_WS (16)	Temp (15)		
O/E_50_	0.75	9	SLOPE (27)	PPT (26)	Temp (21)	IMPERVMEAN_WS (14)	MAFLOWU (12)		
Sierras (*n* = 203)									
O/E	0.51	13	SLOPE (36)	PPT (22)	Temp (20)	rDDENSC12_WS (12)	NewUrb_5km (10)		
O/E_50_	0.60	30	PPT (42)	SLOPE (24)	rDDENSC12_WS (12)	GrAZING_WS (12)	Temp (10)		
Miscellaneous group									
Central Valley (*n* = 54)									
O/E	0.64	4	CondQrm (64)	SLOPE (25)	AgUrb21_1km (6)	Temp (5)			
O/E_50_	0.44	4	CaO (26)	SLOPE (24)	Temp (22)	CondQrm (14)	PctSed_ws (7)	Ag_WS (7)	
Interior Chaparral (*n* = 75)									
O/E	0.65	26	SLOPE (35)	CondQrm (29)	IMPERVMEAN_WS (27)	Ag_WS (9)			
O/E_50_	0.71	5	PPT (39)	CondQrm (35)	IMPERVMEAN_1km (26)				
Modoc (*n* = 56)									
O/E	0.64	7	CondQrm (26)	Ag_WS (21)	Temp (20)	IMPERVMEAN_WS (18)	SLOPE (15)		
O/E_50_	0.66	3	CondQrm (29)	IMPERVMEAN_WS (26)	Ag_WS (23)	Temp (22)

Regional response models (Table 3) were broken up into three groups: a coastal group (South Coast, Coastal Chaparral, and North Coast), a mountain group (Lahontan and Sierras), and a remaining group of regions with mixed responses and smaller sample sizes (Interior Chaparral, Central Valley, and Modoc). All the regions in the coastal group had some measure of human disturbance as the variable with highest relative importance (Table 3). The South Coast and Coastal Chaparral region models included measures of population density or urban land cover within 1 or 5 km as the variable with the highest relative importance. The North Coast region models were characterized by AGURB21 at the watershed scale, site elevation, and slope (Table 3). Thus, stream biological condition in the South Coast and Coastal Chaparral appeared to be sensitive to local human disturbance, but in the North Coast region, biological condition was more affected by human disturbance at the watershed scale.

**Fig. 4:**
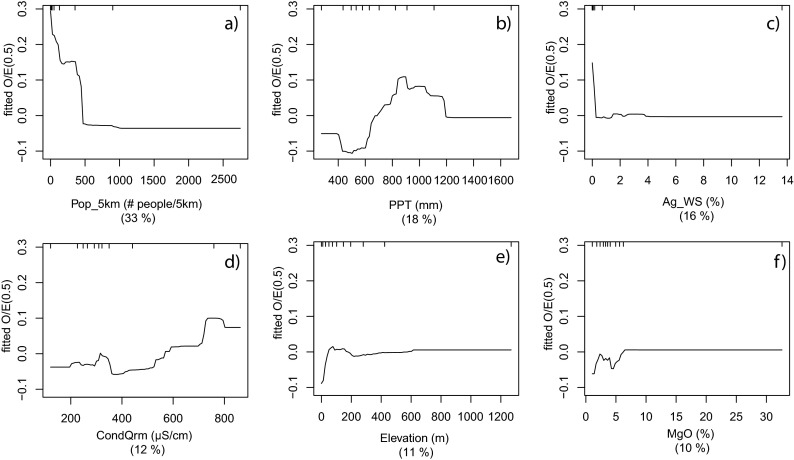
Partial dependency plots for the final Coastal Chaparral model (*n* = 216) of the response form of O/E_50_ (*y*-axis = fitted function of O/E_50_) based on the effect of individual explanatory variables with the response of all other variables removed. Variables are shown in order of model importance: **a** population density in 2000 at 5 km scale (Pop_5km); **b** average precipitation (PPT); **c** agricultural land use at watershed scale (Ag_WS); **d** predicted conductivity (CondQrm); **e** average temperature (Temp); and **f** Percent magnesium oxide mineral content (MgO). The *top tick marks* of each plot indicate deciles of the predictor variable

The Coastal Chaparral models had the highest *R* ^2^ of all models (Table 3). Partial dependency plots for the variables in the O/E_50_ model for this region show a variety of responses. The most important variable, population density at the 5 km scale (Pop_5km), showed an immediate approximately linear decline in the response variable as Pop_5km increased to about 500 persons/km^2^ (Fig. 4a). A rapid decline was also apparent for percentage agriculture in the watershed (Ag_WS) (Fig. 4c); however, the entire response occurred over a very narrow range of values (<4 %). Of the remaining variables associated with O/E_50_, the forms of the responses were mixed (Fig. 4b, d–f).

**Fig. 5:**
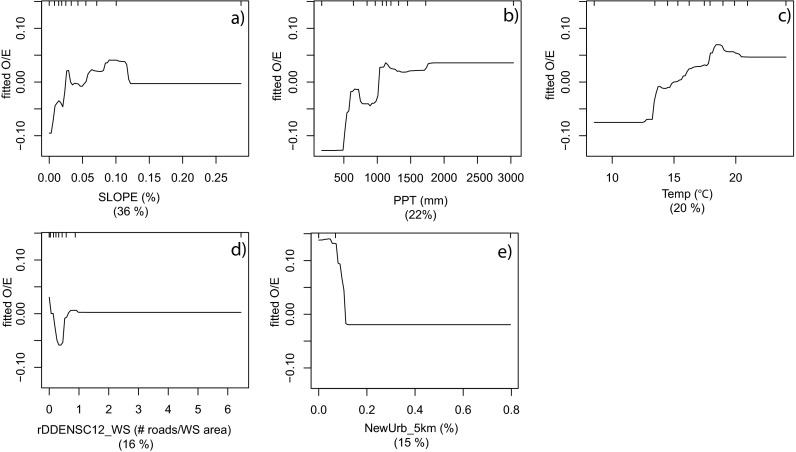
Partial dependency plots for the final Sierra region (*n* = 203) of the response form of O/E (*y*-axis = fitted function of O/E_50_) based on the effect of individual explanatory variables with the response of all other variables removed. Variables are shown in order of model importance: **a** slope; **b** average precipitation (PPT); **c** average temperature (Temp); **d** total density of paved roads at watershed scale (rDDENSC12_WS); and **e** percent new urban at 5 km scale (NewUrb_5km). The *top tick marks* of each plot indicate deciles of the predictor variable

The mountain group (Lahontan and Sierras) models were strongly influenced by variables representing natural environmental gradients, including stream slope and climatic variables (Table 3). At least one measure of human disturbance was included in each of the models but the relative importance of these variables never exceeded 17 (Table 3). The Sierras partial dependency plots suggest that O/E was positively associated with relatively steep, warmer streams in areas of higher precipitation within the Sierras region (Fig. 5a–c). Human disturbance variables had lower relative importance than the natural gradients; however, the limited responses to human disturbance appeared to occur at very low levels of disturbance (Fig. 5d–e).

The remaining three regions gave variable results, probably due to small sample sizes and low reference site representation (Table 3). The O/E and O/E_50_ models within each region often included different variables or the same variables with varying relative importances. The Modoc region is a higher elevation region with little disturbance and much of the Interior Chaparral Region is privately owned ranch land. The Modoc and Interior Chaparral models are probably reflecting a mix of natural and human disturbance factors. The Central Valley has been extensively utilized for intensive agriculture for many years. The low relative importance of human disturbance variables is likely the result of there being few reference sites and all the remaining sites being highly disturbed (Table 2). The Central Valley models may be demonstrating the effects of variation in natural gradients, such as soil characteristics, slope, and geology, within a highly human-modified ecosystem. The variables in these three regions generally have similar relative importances among the top 2 or 3 variables, suggesting there are no strong environmental gradients for either natural variables or measures of human disturbance within the sites available from those regions (Table 3).

**Fig. 6:**
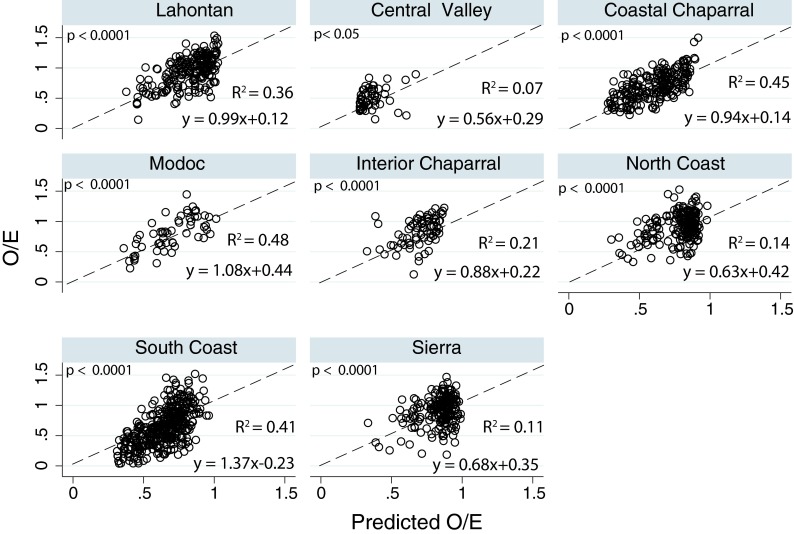
Regional regressions and plots of observed O/E values as a function of O/E values predicted using the statewide model. The *dashed line* represents the 1:1 line

The statewide models had variable success predicting O/E and O/E_50_ for individual regions (Fig. 6). All regional regressions were statistically significant. With the exceptions of the North Coast and Sierras, the 95 % confidence interval for the slope coefficients included the 1:1 line; however, the 95 % confidence interval was broad for the Central Valley (0.06–1.06) and Interior Chaparral (0.49–1.26), as reflected in the low *R* ^2^. The statewide model was reasonably successful at predicting Coastal Chaparral O/E scores, and predictions had minimal bias based on visual inspection (Fig. 6). The statewide model was also reasonably successful at predicting South Coast O/E scores; however, the model appeared biased toward over-predicting at sites with low O/E scores and under-predicting at sites with higher O/E scores. Within the Coastal Group, the statewide model was least successful at predicting North Coast O/E scores (Fig. 6). The North Coast had few observed values of O/E less than about 0.7. Similar to the North Coast, the Lahontan and Sierras had few observed O/E values less than about 0.8 (Fig. 2). The statewide model was reasonably successful at predicting Lahontan model O/E scores (Fig. 6) but slightly less successful than for the South Coast and Coastal Chaparral. There was no clear bias in the predicted Lahontan values. The statewide model was not successful at predicting Sierra O/E scores. This difference between the two Mountain Group regions might be due to the Sierras having fewer sites with low to moderate O/E scores. The statewide model was variably successful at predicting O/E scores for the remaining groups with smaller sample sizes (Interior Chaparral, Central Valley, and Modoc) (Fig. 6). The statewide model was reasonably successful at predicting Modoc O/E scores and this regression had the highest *R* ^2^ of all the regions. The Interior Chaparral region had several values that were well off the 1:1 line and at the higher observed values the statewide model appeared to under-predict values. The Central Valley region was notable because of the extremely narrow range of observed values, and the generally low O/E observed values with only three sites greater than 0.7. While the relationship between Central Valley region and the statewide model was significant, the statewide model was a poor predictor of O/E scores in the Central Valley.

## Discussion

The primary objective of this study was to construct BRT models of macroinvertebrate taxonomic completeness (as measured by O/E and O/E_50_) in relation to commonly available GIS-derived variables for the state of California and regions within the state. Overall, we were able to develop significant BRT models for the state and all PSA regions and have confidence in the regional differences highlighted by our analyses. The models derived here were intended to identify important predictor variables at the statewide and regional levels to increase understanding of California streams and to aid state management agencies as numerical biocriteria are developed. Clearly, larger sample sizes provide higher confidence in the final BRT models and the applicability of our results for aiding management and prioritizations efforts.

While we were able to derive significant BRT models for the entire state and all regions, the regional models for Modoc, Interior Chaparral, and Central Valley were limited by small sample size and few identifiable reference sites. Models for O/E_50_ typically had higher *R* ^2^ values than models for O/E providing support for the assumption that models for predicting numbers of expected taxa based on taxa with PC >0.5 have lower variance than models including rare taxa (Ostermiller and Hawkins 2004; Van Sickle et al. 2007). The Central Valley and South Coast regions were exceptions with *R* ^2^ for the O/E model slightly higher than for O/E_50_. This might suggest that the expected reference condition in these regions may be defined on the basis of taxa that are always rare, even at sites with good biological condition. An alternative explanation is that the responsiveness of rare taxa outweighs the overall variance reduction gained by using PC >0.50.

Statewide models for O/E and O/E_50_ explained at least 50 % of the variation in taxonomic completeness and included the same variables with the composite measure of human disturbance at the 1-km scale (AGURB21_1km) being most important (Table 3). The partial dependency plot (Fig. 3a) showed an approximately linear decline in taxonomic completeness as AGURB21_1km increased. The usefulness of composite disturbance metrics has been noted by others and such metrics have been used for planning biodiversity conservation in many areas of the world (Brown and Vivas 2005). Our results suggest that predictive models of biological conditions at unsampled sites could be developed at the statewide level; however, our regional results suggest that such predictions would have limited utility in some regions, such as the North Coast and Sierra (Fig. 6). We chose not to develop validated, predictive models in this study because several of the regions had insufficient sample sizes to create the needed development and validation data sets. Our results identified several regions of the state where additional sampling would likely improve both regional and statewide models.

The models for the coastal group were strongly influenced by measures of human disturbance. For South Coast and Coastal Chaparral models, local watershed measures of urbanization (Urban_1 and Pop_5km, respectively) were most important. These results suggest that human disturbance in the nearby portion of the watershed is a better predictor of taxonomic completeness than watershed level disturbance. This is not surprising because of the patterns of development in these two regions, which include the largest and most populous areas in the state—the San Francisco Bay, Los Angeles, Orange County, and San Diego metropolitan areas. Intensive development in these areas typically occurs as streams enter lower gradient sections and slopes decrease allowing for development in a relatively flat landscape (Brown et al. 2012). The upper portions of the watersheds are often included in open space or protected areas (Brown et al. 2012). Thus, stream degradation can occur rapidly as streams enter areas where intensive development is possible and near-site indicators may better capture this process.

Brown et al. (2012) developed a BRT model for IBI scores for 159 sites from a region of Southern California which corresponds to our South Coast and Coastal Chaparral regions combined. The most important variables in their model were population density within the watershed, agricultural plus urban land use in the riparian zone, the density of man-made channels, and the mean annual precipitation (*R* ^2^ = 0.66). IBI scores and O/E scores are correlated (Ode et al. 2008, 2011), indicating that comparisons between these studies are reasonable. The reason for Brown et al. (2012) finding a watershed scale variable most important may relate to methods of measurement. Brown et al. (2012) utilized local watershed variables in the form of a 90-m buffer strip to both sides of stream centerlines. The sampling programs utilized in both studies generally avoided highly artificial streams (i.e., channels with concrete bottoms) but sampled channelized streams with natural bottoms, many of which had vegetated floodplain areas to accommodate high storm flows. While the buffer method might be appropriate for agricultural streams where land is tilled to the edge of the active stream channel or highly channelized streams in urban areas, it likely underestimates near-stream disturbance in the South Coast and Coastal Chaparral regions. In these regions, the local watershed radial clips (1 or 5 km) are likely a more appropriate measure for assessing local watershed disturbance. Similar to the Coastal Chaparral in this study (Fig. 4a), Brown et al. (2012) noted a rapid decline of biological condition in response to population density. Brown et al. (2012) observed that most of the response occurred at watershed population densities less than 300 persons/km^2^. Thus, the effect of human disturbance appears to occur fairly rapidly in these regions.

In the North Coast region, AGURB21_WS was the most important factor affecting taxonomic completeness. This region lacks large metropolitan areas or extensive agriculture, and the variables reflecting human disturbance include more dispersed factors such as logging, roads, fire, and sedimentation associated with such factors (Short 2013). Thus, it was not surprising that a watershed-level measure of human disturbance was most important. We included variables such as road density and fire characterization (number of burns) but these variables were never given high importance in our models. Variables such as these that contribute to non-point source problems, such as sedimentation, and that may have cumulative effects are often difficult to characterize from existing GIS coverages. The effects of logging and fire also change over time as vegetation recovers and soils stabilize (Keeley 2009), which is difficult to capture from static GIS coverages. It is significant that a human disturbance variable proved to be important in the model. Rehn et al. (2005) developed an IBI for the North Coast region and found that the community attributes associated with IBI scores were responsive to watershed disturbance measures.

In the models for the mountainous Lahontan and Sierras regions, natural gradients of slope, precipitation, and temperature were more important than measures of human disturbance. The dominance of natural predictors in the models implies that these regions, as a whole, are relatively unmodified by human activities. However, we are not suggesting that human disturbance does not affect streams in these regions. We suspect that our data set did not contain a sufficient number of moderately to highly disturbed sites (Figs. 2 and 6) to characterize the effects of human activity. The data sets for both regions were heavily weighted toward reference sites with 59 % reference sites for Lahontan and 58 % reference sites for the Sierras. Studies in these regions that were focused on evaluating different types of human disturbances have documented the specific effects of hydrologic modification (Rehn 2008; Yarnell et al. 2010) and logging (Hawkins et al. 2000; Herbst and Silldorff 2006; Herbst et al. 2012) on aquatic biota. Developing a predictive model for these regions that incorporates the effects of human disturbance will likely require data from targeted studies designed to characterize such relations.

While we were able to develop BRT models for the Interior Chaparral and Modoc regions, we believe these models should be treated with caution. The sample sizes were small (*n* ≤ 75 sites) and the importance of the variables was very different between the O/E and O/E_50_ models (Table 3). Additional sampling in these regions will likely be necessary to develop more robust models. This may be particularly urgent for the Interior Chaparral because this region is likely to experience rapid development as Central Valley communities expand into the foothill regions (Sleeter et al. 2011). This region has a very diverse aquatic fauna and is a vital corridor between the Sierra Nevada and Central Valley regions and are deserving of protection (May and Brown 2002; Ode et al. 2011).

The same cautions applied to the Interior Chaparral and Modoc regions apply to models for the Central Valley. It was surprising that measures of agricultural land use had little importance in this region because Central Valley sites had the highest median human disturbance of all of the regions (Table 2), primarily due to agricultural activities. The Central Valley is problematic because all the sites are highly disturbed (with only 1 of the 54 sites classified as “reference”). Most of the variability among sites appears to be related to the interaction of the agricultural landscape with natural factors, such as slope and predicted conductivity (Table 3). Also, because of the disturbed nature of the landscape, no Central Valley sites were included in the development of the O/E models used to calculate expected values (Ode et al. 2011). Thus, we cannot be sure if the O/E models are based on appropriate expected values for this region. This lack of reference conditions will likely require some alternative approaches for determining appropriate biological objectives for this region (Ode et al. 2005b; Rehn et al. 2008). For example, Carter and Fend (2005) suggested a factor-ceiling approach for determining the best attainable or reference condition for highly urbanized areas like the San Francisco Bay area.

Regions that included a measure of human disturbance in the regional model were generally well-fit by the statewide model, in terms of their visual fit and *R* ^2^ (Fig. 6). The variable AGURB21_1km appears to be a good composite metric for summarizing the effects of human disturbance on stream systems in California. The statewide model may serve as a preliminary assessment tool when addressing questions at large geographic scales, such as national or continental scales, but caution is necessary for regions that were not well fit by the statewide model. Additional sampling and modeling in those regions are necessary before the overall utility of a statewide model can be determined.

It seems likely that regional models will be necessary for understanding the variables associated with stream condition in different areas of the state. The general concept that smaller scale regional models perform better than larger scale model has been supported by other researchers. Ode et al. (2008) found that macroinvertebrate indices developed at large regional scales such as the western USA had lower precision in California compared to California-based indices. They found that larger scale indices were influenced by two natural gradients (percent slope and percent fast water habitat) that did not affect the statewide indices. Overall, national- or large-scale models are likely to focus on large-scale natural environmental setting variables such as climate and geology as the primary discriminating variables with disturbance variables such as land use, nutrients, fine sediments, and contaminants as secondary variables. For Portuguese streams, Feio et al. (2009) found that regional O/E models predicted more taxa than the national model, were more accurate, and had lower misclassification error rates when placing sites into pre-defined groups. The regional models were also more sensitive to some disturbances related to water chemistry and land use. In streams of the Northeastern USA, Waite et al. (2014) found that large-scale models of macroinvertebrate metrics explained nearly as much variance in the macroinvertebrate data as smaller scale individual ecoregion models. They suggest that it may be advantageous for bioassessment programs to develop large regional models (in their case multi-ecoregion models) as a preliminary assessment of overall disturbance conditions as long as the range in natural landscape variability can be minimized. Smaller scale regional models can then be developed to refine understanding and improve predictive power of the models.

Environmental and resource protection agencies worldwide are given the tasks of protecting biodiversity and other natural resources. These tasks require a wide range of available tools to be successful (Barmuta et al. 2011). California water quality and environmental agencies are currently developing numerical biological objectives for water quality management and anti-degradation policies to protect pristine areas (Ode et al. 2011). The development of models such as ours is an important step in understanding the types and spatial scale of human disturbances that are associated with stream biological condition in any geographic region (Mattson and Angermeier 2007). Development of validated models suitable for predicting biological condition at unsampled sites is a valuable next step, given adequate data. Such models can be extremely useful tools in the protection and conservation of biodiversity and aquatic resources.

## Conclusions

We successfully constructed significant BRT models of macroinvertebrate taxonomic completeness (as measured by O/E) in relation to commonly available GIS-derived variables for the state of California and regions within the state. Surprisingly, the statewide models described almost as much variance (i.e., *R* ^2^) as some of the regionally specific models. The variable AGURB21_1km appears to be a good composite metric for summarizing the effects of human disturbance on stream systems in California; however, at the regional scale, more specific measures of disturbance or natural gradients were more important. The statewide models had variable success predicting O/E for individual regions. As new data become available, refined models can be developed and increased sample sizes will allow for development of predictive BRT models. The understanding gained from the models presented here can be useful for general applications such as identifying and prioritizing regions for monitoring, remediation, or preservation for anti-degradation actions, stratifying new bioassessments according to anticipated biological condition, or assessing the potential for change in stream taxonomic completeness based on anticipated changes in land use and land cover.

## Appendix

**Table 4:** Geographic information systems sources and references

Spatial dataset	Data source	Source data format	Processing format	Resolution	Reference
Hydrography	National Hydrography Dataset (NHD)	Vector	Vector	1:24,000	U.S. Geological Survey, National Hydrography Dataset, Digital data, accessed, January 2010 at http://nhd.usgs.gov/data.html
Land Cover 1992	National Land Cover Dataset 1992 (NLCD)	Raster	Vector	30 m	U.S. Geological Survey, National Land Cover Dataset, Digital data, accessed, January 2010 at http://landcover.usgs.gov/
Land Cover 2001	NCLD 2001	Raster	Vector	30 m	U.S. Geological Survey, National Land Cover Dataset, Digital data, accessed, January 2010 at http://www.mrlc.gov/
Elevation	National Elevation Dataset (NED)	Raster	Raster	10 m	U.S. Geological Survey, National Land Cover Dataset, Digital data, accessed, January 2010 at http://seamless.usgs.gov/
Slope	NED	Raster	Raster	10 m	U.S. Geological Survey, National Land Cover Dataset, Digital data, accessed, January 2010 at http://seamless.usgs.gov/
Roads	U.S. Census Bureau Tiger	Vector	Vector	1:100,000	U.S. Census Bureau, TIGER line data, Digital data, accessed, January 2010 at http://www.census.gov/geo/www/tiger/
Population density	U.S. Census Bureau 2000	Vector	Raster	30 m	U.S. Census Bureau, Census 2000, Digital data, accessed, January 2010 at http://www.census.gov/main/www/cen2000.html
Precipitation and temperature	Oregon State University PRISM	Raster	Raster	30 arc-seconds	Parameter-elevation relationships on Independent Slopes Model Group, Oregon State University, Precipitation and Temperature data for the USA, digital data, accessed, January 2010 at http://prism.oregonstate.edu/
Dams	National inventory of dams	Vector	Vector	Various	U.S. Army Corps of Engineers, National Inventory of Dams, digital data, not publicly available
